# Interstitial lung disease occurring shortly after tocilizumab infusion in a patient with polyarticular juvenile idiopathic arthritis: a case report

**DOI:** 10.1186/s13223-021-00594-7

**Published:** 2021-09-08

**Authors:** Koichi Sugihara, Risa Wakiya, Hiromi Shimada, Mikiya Kato, Tomohiro Kameda, Shusaku Nakashima, Mai Mahmoud Fahmy Mansour, Yusuke Ushio, Norimitsu Kadowaki, Hiroaki Dobashi

**Affiliations:** Division of Hematology, Rheumatology and Respiratory Medicine, Department of Internal Medicine, Faculty of Medicine, Kagawa University, 1750-1 Ikenobe, Miki-cho, Kita-gun, Kagawa 761-0793 Japan

**Keywords:** Juvenile idiopathic arthritis, Drug-induced interstitial lung disease, Tocilizumab, Interleukin-6

## Abstract

**Background:**

Tocilizumab has been shown to be effective for treatment of juvenile idiopathic arthritis (JIA). To our knowledge, this is the first reported case of interstitial lung disease occurring shortly after tocilizumab infusion in a patient with JIA.

**Case presentation:**

A 14-year-old female patient with polyarticular JIA developed interstitial lung disease after intravenous and subcutaneous administration of tocilizumab. Her condition improved with glucocorticoid therapy.

**Conclusion:**

Our results suggest that increased interleukin-6 levels in the blood following tocilizumab treatment may be linked to development of interstitial lung disease.

## Background

Juvenile idiopathic arthritis (JIA) is chronic arthritis of unknown cause that occurs in individuals under 16 years of age and persists for at least 6 weeks. Pro-inflammatory cytokines such as interleukin (IL)-1, IL-6, IL-18 and tumor necrosis factor-α play important roles in the pathogenesis of JIA [1, 2].

Tocilizumab is a humanized anti-IL-6 receptor antibody developed in Japan. The drug has been shown to be effective for JIA and is approved in Japan for treatment of systemic and polyarticular JIA recalcitrant to conventional therapies [3].

Various adverse events, including infections and infusion reactions, have been observed in JIA patients treated with tocilizumab. However, no adverse events with pulmonary involvement have been reported [4]. Herein, we report the case of a patient with polyarticular JIA who developed interstitial lung disease following tocilizumab treatment.

## Case presentation

A 14-year-old female patient with a 4-month history of arthralgia was referred to our institution from an orthopedic clinic in April. She had undergone surgery for patent foramen ovale at age 3 and she has had anemia (hemoglobin of 60-80 g/L) since age 7. Several examinations for anemia including bone marrow puncture performed in the pediatric department of another hospital did not identify the cause of anemia. She has received treatment such as iron replacement and red blood cell transfusions when the anemia worsened. The patient had no significant familial medical history. She was diagnosed with polyarticular rheumatoid factor-positive JIA according to the International Federation of Rheumatology's classification criteria [5] (presence of arthritis in five or more joints within 6 months of onset and two or more positive rheumatoid factors measured at intervals of 3 months or longer). The patient was administered naproxen, but her active arthralgia persisted. She received an intravenous infusion of 8 mg/kg tocilizumab for ongoing arthralgia with active synovitis on admission and was discharged 3 days after the first infusion because no adverse events occurred. However, she developed dyspnea 2 days after discharge and was re-admitted.

The patient’s percutaneous oxygen saturation was 55% on room air and 94% on 3 L/min of nasal oxygen. She was afebrile, and her blood pressure and pulse rate were normal. The eyelid conjunctiva were pale. On auscultation, fine crackles were heard in bilateral lung fields and a systolic murmur of Levine grade 1 was heard in the second intercostal space of the right margin of the sternum. Joint findings showed tenderness and swelling of the left mandibular joint, bilateral shoulder joints, bilateral wrist joints, bilateral ankle joints, right second and third metatarsophalangeal joints, and left first and fourth metatarsophalangeal joints.

The patient’s laboratory findings are summarized in Table 1. She had anemia (hemoglobin of 59 g/L). Lactate dehydrogenase, Krebs von den Lungen-6 and ferritin were elevated to 612 IU/L, 1820 U/mL and 593.7 µg/L, respectively. Her IgG was elevated to 21,490 mg/L. All tests for infectious diseases were negative.

**Table 1:** Laboratory test results 5 days after the first tocilizumab administration

Hematology		Biochemistry		Immunology	
WBCs	5.76 × 10^9^/L	CRP	0.1 mg/L	ANA	× 40 (homo)
Neutrophils	73.0%	TP	71 g/L	IgA	1970 mg/L
Eosinophils	1.0%	Alb	36 g/L	IgM	1370 mg/L
Basophils	1.0%	Urea	3.6 mmol/L	IgG	21,490 mg/L
Lymphocytes	21.0%	Cr	31 µmol/L	β-D glucan	−
Monocytes	4.0%	T-Bil	14 µmol/L	Procalcitonin	−
RBCs	2390 × 10^9^/L	AST	38 IU/L	T-SPOT	−
Hb	59 g/L	ALT	19 IU/L	CMV Ag (C10, 11)	(0,0)
Hct	19.9%	ALP	246 IU/L	Anti-*Trichosporon asahii* antibody	−
Platelets	231 × 10^9^/L	LDH	612 IU/L	Blood gas analysis (nasal oxygen 3 L/min)	
MCV	83.3 fL	γ-GTP	14 IU/L	pH	7.429
MCH	24.7 pg	Na	136 mmol/L	PaO_2_	71.1 mmHg
MCHC	29.6%	K	4.2 mmol/L	PaCO_2_	33.1 mmHg
Ret	10.1%	Cl	105 mmol/L	HCO_3_	21.4 mmol/L
		KL-6	1820 U/mL	BE	-2.6 mmol/L
		Ferritin	593.7 µg/L		

*WBC* white blood cell, *RBC* red blood cell, *Hb* hemoglobin, *Hct* hematocrit, *MCV* mean corpuscular volume, *MCH* mean corpuscular hemoglobin, *MCHC* mean corpuscular hemoglobin concentration, *Ret* reticulocytes, *CRP* C-reactive protein, *TP* total protein, *Alb* albumin, *Cr* creatinine, *T-Bil* total bilirubin, *AST* aspartate aminotransferase, *ALT* alanine aminotransferase, *ALP* alkaline phosphatase, *LDH* lactate dehydrogenase, *γ-GTP* gamma glutamyl transferase, *KL-6* Krebs von den Lungen-6, *ANA* anti-neutrophil antibody, *T-SPOT* T-SPOT®.*TB* test for *Mycobacterium tuberculosis*, *CMV Ag* cytomegalovirus antigen, *BE* base excess

**Fig. 1:**
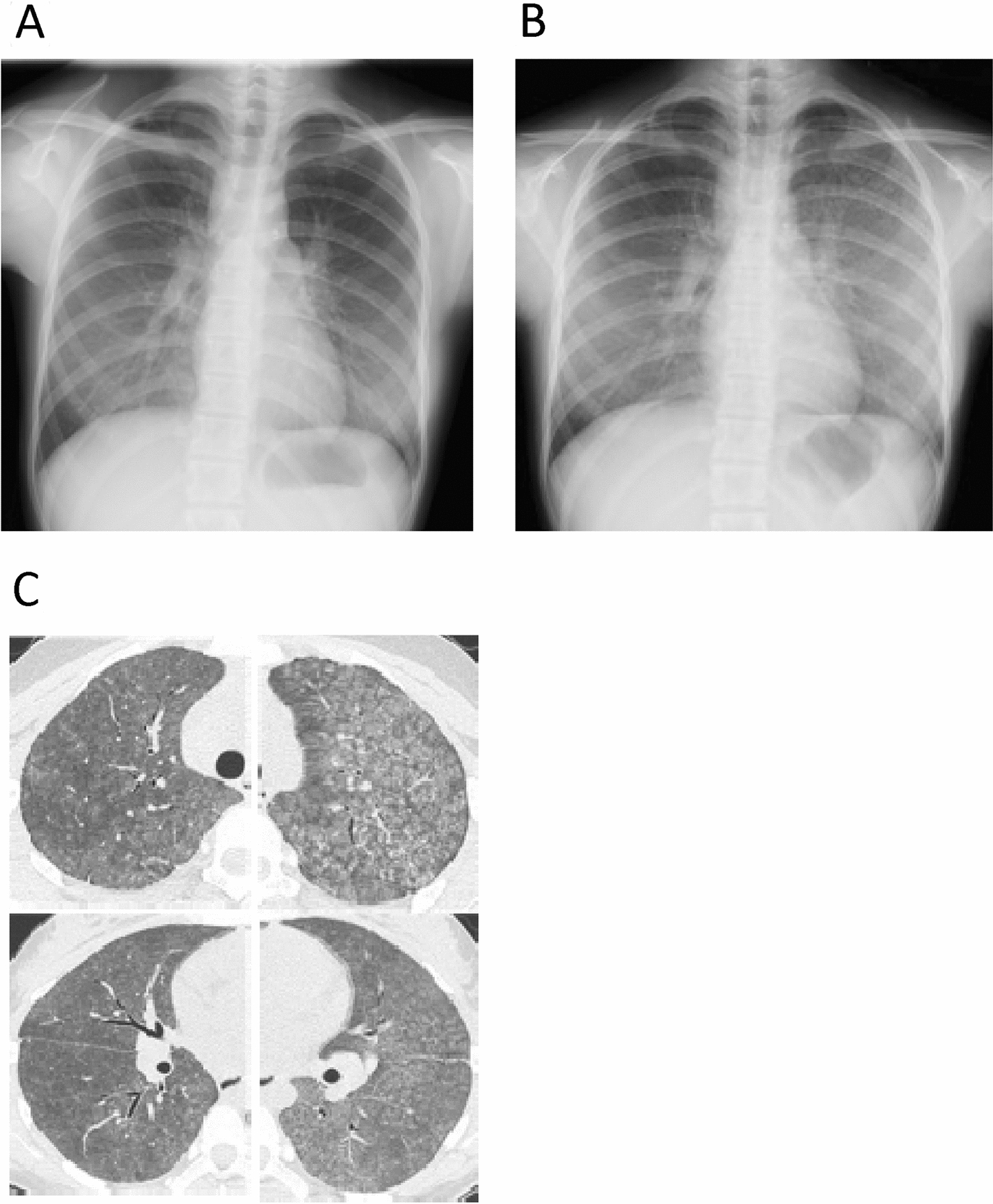
Chest X-ray images before and after tocilizumab administration and chest CT image after tocilizumab administration. Chest X-ray images before (**A**) and 5 days after (**B**) tocilizumab administration and chest CT image 5 days after tocilizumab administration (**C**)

Chest X-ray showed ground-glass opacities bilaterally (Fig. 1). A computed tomography (CT) scan revealed a broad range of panlobular or acinar ground-glass opacities (Fig. 1). Bronchoalveolar lavage was not performed due to severe dyspnea. Because of her clinical course (dyspnea appearing immediately after discharge) and test results including CT findings, we diagnosed the patient with hypersensitivity pneumonitis and prescribed prednisolone (30 mg/day) without antibiotics. She was discharged 4 days after admission because her dyspnea and imaging findings improved immediately after the initiation of glucocorticoid treatment. Anti-Trichosporon asahii antibody was not detected.

**Fig. 2:**
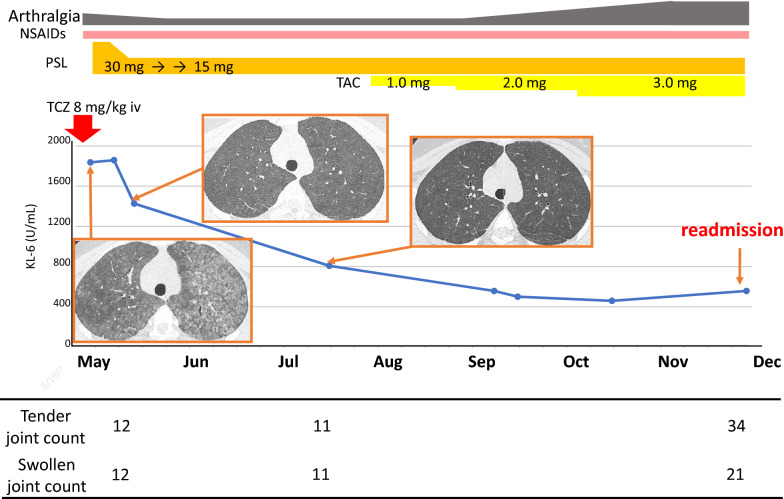
Clinical course of the patient after the first tocilizumab administration. *NSAIDs* non-steroidal anti-inflammatory drugs, *PSL* prednisolone, *TCZ* tocilizumab, *TAC* tacrolimus, *KL-6* Krebs von den Lungen-6, *iv* intravenous

**Fig. 3:**
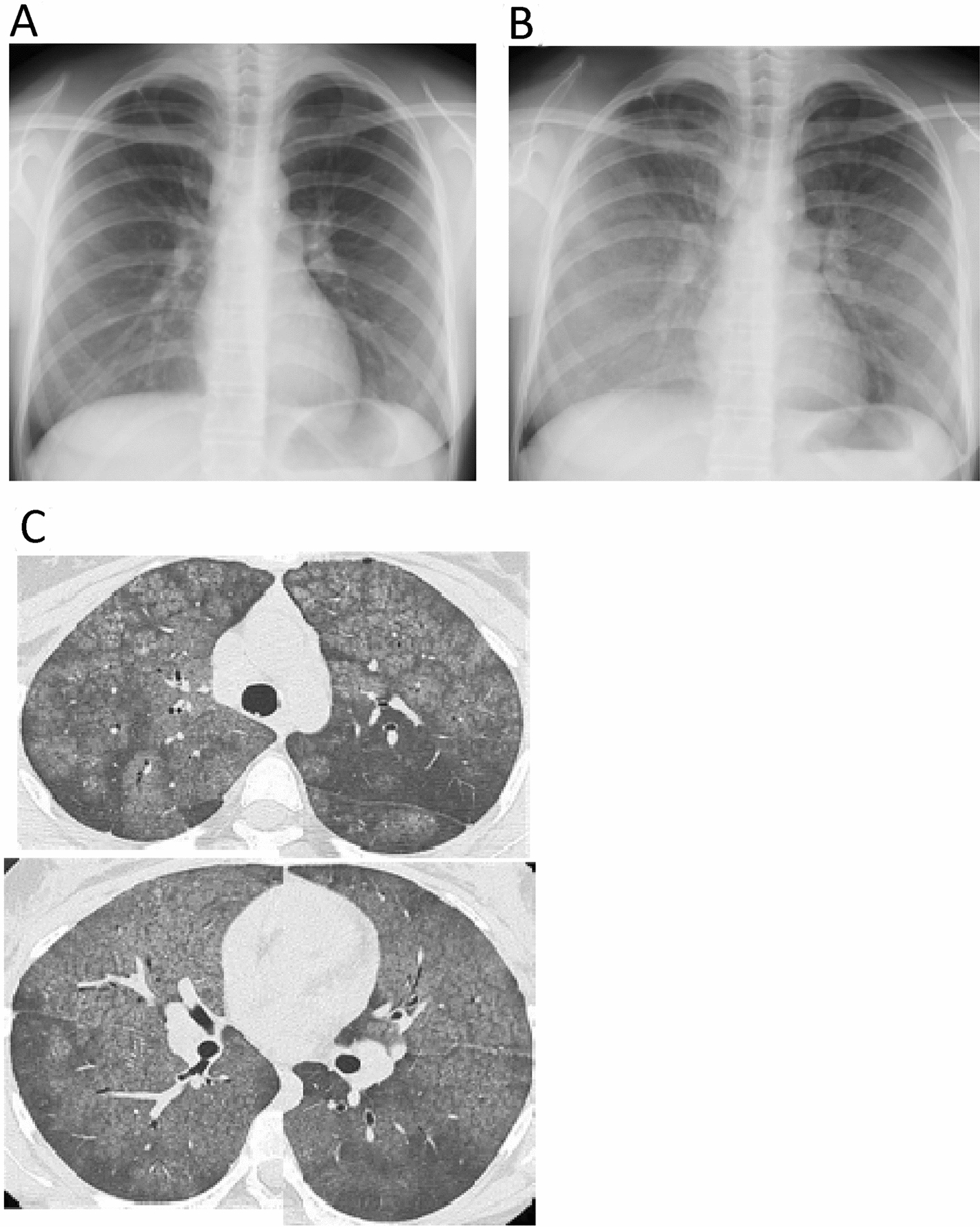
Chest X-ray images and chest CT image after tocilizumab re-administration. Chest X-ray images 8 (**A**) and 11 (**B**) days after tocilizumab re-administration and chest CT images 11 days after tocilizumab re-administration (**C**)

The dose of prednisolone was decreased gradually and tacrolimus was subsequently added to her regimen. However, she was re-admitted 6 months after discharge to receive additional tocilizumab because her arthritis deteriorated under a reduced dose of prednisolone. We considered the hypersensitivity pneumonitis with the first treatment was more likely caused by house antigens such as fungus, house dust or bird droppings other than tocilizumab, because it occurred at home after discharge from the hospital. In addition, the efficacy of tocilizumab on arthritis was observed after the first tocilizumab infusion. These are why tocilizumab was reintroduced. Eight days after the injection, she was discharged from hospital with no recurrence of lung lesions. However, 11 days after the injection, she again developed dyspnea and she was urgently hospitalized with hypoxia (Fig. 2). Imaging findings were like those after initial administration of tocilizumab, with extensive ground-glass opacities visible on chest X-ray and chest CT (Fig. 3).

**Fig. 4:**
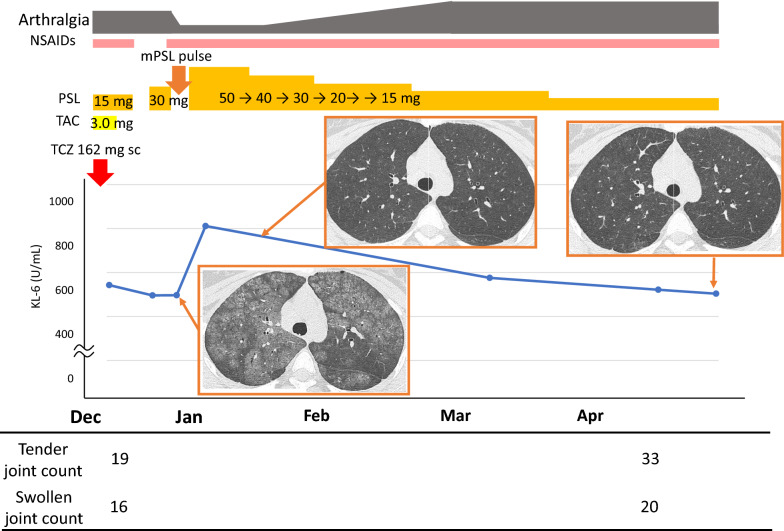
Clinical course of the patient after tocilizumab re-administration. *NSAIDs* non-steroidal anti-inflammatory drugs, *PSL* prednisolone, *mPSL* methylpredonisolone, *TCZ* tocilizumab, *TAC* tacrolimus, *KL-6* Krebs von den Lungen-6, *sc* subcutaneous

Prednisolone (30 mg daily) was started again, but the patient’s hypoxia progressed and imaging findings showed no improvement. Methylprednisolone (1 g/day) was administered as pulse therapy for 3 days, followed by prednisolone (50 mg/day). As her dyspnea and chest imaging findings improved, prednisolone was decreased gradually. The ground-glass opacities visible on chest CT disappeared and she was discharged 20 days after admission (Fig. 4). A drug-induced lymphocyte stimulation test was conducted for tocilizumab but was negative. Because of a flare-up of polyarthritis associated with steroid tapering, etanercept (50 mg/week) was introduced 4 months after discharge in May of the following year. After introduction of etanercept, the patient’s polyarthritis improved. No recurrence of interstitial lung lesions has been observed as of October of the following year despite a reduction of her daily dose of prednisolone to 3 mg.

## Discussion and conclusions

The patient with JIA described in this report developed pulmonary lesions after both intravenous and subcutaneous injections of tocilizumab. We hypothesized that pulmonary involvement in this case was a complication of JIA, infection, or the drugs administered. Generally, 10–20% of patients with rheumatoid arthritis have pulmonary involvement, but patients with JIA rarely develop lung lesions [6]. The case described here showed no pulmonary lesions prior to tocilizumab treatment.

Maynart et al. reported nonspecific interstitial pneumonia developed in refractory systemic JIA and it was responded to tocilizumab treatment [7]. Kimura et al. reported that pulmonary hypertension, alveolar proteinosis and interstitial lung lesions were complications of systemic JIA and suggested a potential association between biologic therapy and pulmonary complications [8]. The patient described in this report had polyarticular JIA, and it is unlikely that pulmonary involvement would have spontaneously and repeatedly occurred as a complication of JIA following tocilizumab administration.

In our case, all laboratory tests of infection-related parameters were negative. The patient’s pulmonary lesions improved with glucocorticoid therapy without antibiotics. Thus, infection was unlikely to be the cause of the lung lesions observed in our case.

Interstitial lung disease was reported as an adverse event occurring among 0.42% of rheumatoid arthritis patients in all-case surveillance of tocilizumab in Japan [4]. In the postmarketing surveillance of tocilizumab for rheumatoid arthritis, interstitial lung disease was reported in 23 patients (0.59%) [9]. Kawashiri et al. and Wendling et al. reported exacerbation of interstitial lung disease during tocilizumab therapy for rheumatoid arthritis [10, 11]. Gouveia et al. and Ikegawa et al. reported organizing pneumonia induced by tocilizumab in a patient with rheumatoid arthritis [12, 13]. On the other hand, no interstitial lung disease has been reported among JIA patients in the surveillance of tocilizumab [4]. However, we consider that our case developed tocilizumab-induced lung disease based on the drug-induced lung injury diagnosis criteria outlined by Tamura et al. [14]. Lung disease occurred following administration of tocilizumab and reoccurred with readministration.

Drug-induced lung injuries are defined as respiratory disorders occurring during the administration of a drug and causally associated with the drug [15]. In general, the mechanisms of drug-induced lung injury can be broadly divided into cytotoxic lung injury, in which the drug itself directly damages lung tissue, alveolar epithelium, airway epithelium or vascular endothelium, and allergic lung injury [16]. Tocilizumab is a humanized monoclonal antibody that is unlikely to have a direct cytotoxic effect on lung tissue, and no such effects have been documented in any report. However, hypersensitivity reactions to tocilizumab in patients with JIA have been reported [17]. JIA patients aged from 2 to 10 years developed fever, abdominal pain, hypotension, and cough minutes to hours after tocilizumab administration, but no cases with only lung injury have been reported. The case documented here was a 14-year-old patient who developed pulmonary lesions 5 and 11 days after tocilizumab administration. Because the patient’s background, time of onset, and symptoms differed from those reported above, her pulmonary lesions were unlikely to be attributable to hypersensitivity reactions. Drug-induced lymphocyte stimulation tests for tocilizumab were negative in our case.

There are several potential mechanisms through which tocilizumab might cause pulmonary lesions. Administration of tocilizumab causes a transient increase in IL-6 levels in the blood; this effect occurs through reduced IL-6 receptor-mediated clearance of IL-6, although production of IL-6 remains unchanged [18]. In our case, blood IL-6 level 18 days before, 5 days after and 222 days after the first tocilizumab infusion was 5.7 pg/mL, 162 pg/mL and 20.1 pg/mL, respectively. On the other hand, IL-6 level on the day of the second infusion (222 days after the first tocilizumab infusion), 13 days after and 126 days after the second infusion was 20.1, 3.6 and 0.6 pg/mL, respectively. There was no data on 11 days after the second tocilizumab infusion when the lung problem reappeared because the patient was treated in a different department. We started glucocorticoid therapy 11 days after the second infusion, so the data on 13 days after the second infusion may have been influenced by the glucocorticoid therapy.

Various reports have revealed the role of IL-6 in the development or exacerbation of lung injury. Nishimoto et al. reported that mice overexpressing IL-6 developed interstitial lung disease [19]. Castleman disease, which causes elevated blood IL-6 levels, is complicated by interstitial lung disease in approximately 60% of patients [20]. Nara et al. suggested that blood IL-6 levels may be a prognostic factor for rapidly progressive interstitial pneumonia associated with clinically amyopathic dermatomyositis [21].

We considered that the changes in IL-6 level after tocilizumab administration was associated with the development of lung disease.

This is the case of lung injury occurring shortly after tocilizumab infusion in a JIA patient. We hope that further research will reveal the molecular mechanism(s) through which excess IL-6 causes lung injury.

## Data Availability

The dataset supporting the conclusions of this article is available upon reasonable request.

## Abbreviations

JIA: Juvenile idiopathic arthritis
IL: Interleukin
CT: Computed tomography
